# Late radiation sequelae as a consequence of breast-conserving therapy with cobalt irradiation aggravated by various risk factors

**DOI:** 10.1259/bjrcr.20150026

**Published:** 2015-05-25

**Authors:** R M Hermann, B Clausing, J Mayer, U M Carl, M Nitsche

**Affiliations:** ^1^Zentrum für Strahlentherapie und Radioonkologie, Bremen/Westerstede, Germany; ^2^Abteilung Strahlentherapie und Spezielle Onkologie, Medizinische Hochschule Hannover, Hannover, Germany; ^3^Department for Thoracic Surgery, Ammerland-Klinik, Westerstede, Germany; ^4^Department for Senology, Ammerland-Klinik, Westerstede, Germany; ^5^Klinik für Strahlentherapie, Karl-Lennert-Krebscentrum, Universität Kiel, Kiel, Germany

## Abstract

This report deals with a 71-year-old female patient who developed cancer in her right breast 20 years ago, underwent breast-conserving surgery and received normofractionated radiotherapy with a ^60^Co unit. 19 years later, fibroids and calcified tissue appeared in her right mammary fold. Furthermore, a deep ulceration developed in this region during chemotherapy of bronchial carcinoma. Apart from being a Type 2 diabetic with arterial hypertension, she was also a habitual smoker. After extensive wound debridement and vacuum-assisted sealing therapy, the affected ribs were dissected and a latissimus dorsi flap was implanted. Our focus here is on the interaction of contributing risks for the development of late radiation sequelae, such as physical (especially unintended hot spots during ^60^Co irradiation) and pathophysiological factors (comorbidities and morbid affections). Fortunately, side-effects such as these are rare nowadays. As this case shows, however, they can be effectively handled by employing modern plastic surgery techniques.

## Clinical presentation

**Figure 1.**
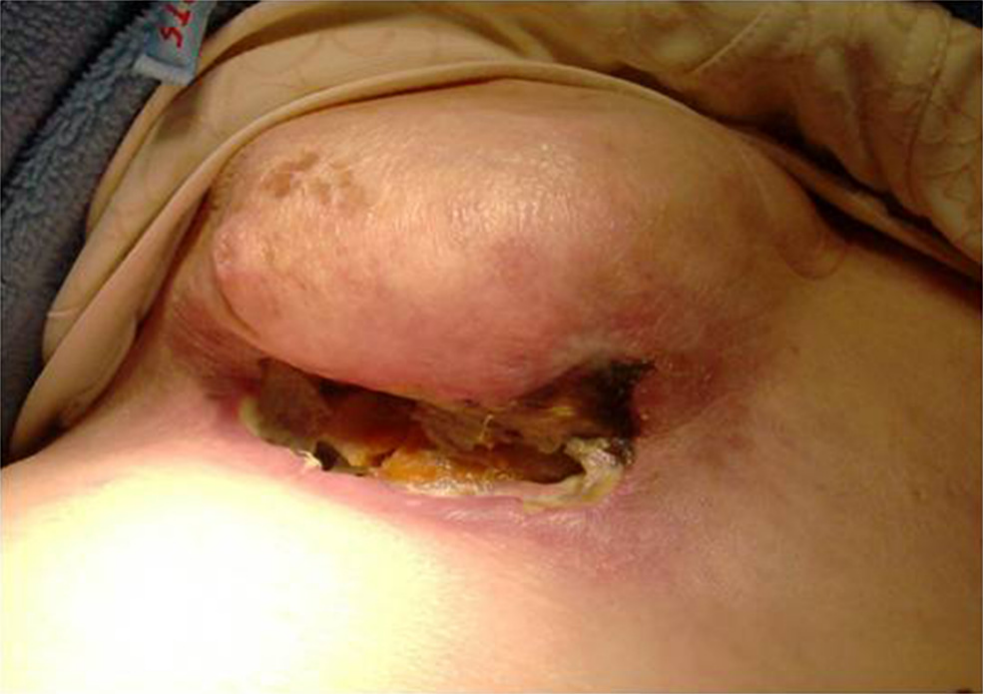
Clinical aspect at first presentation: the complete right breast had hardened and turned into a calculus. Approximately 15 × 5 cm ulceration of the right mammary fold. In the ulceration, a fractured fourth rib and the pleura could be seen. The wound was inflammatory.

A 71-year-old female was referred to our breast cancer department. She suffered from a 15 × 5 cm-sized ulceration that had developed in her right mammary fold together with extensive calcifications (Figure 1). Her Karnofsky performance status was 70%, the general condition impaired. The patient had mild fever (39.1°C). Her right breast had hardened and turned into a calculus. In the ulceration, a fractured fourth rib and the pleura could be seen. The wound was inflammatory. No axillary or supraclavicular lymph nodes were perceptible by palpation.

She reported that the ulceration process had taken place over the past 12 months. Extensive treatment with antiseptic and moistening ointments were of no avail.

The patient had been suffering from metabolic syndrome with arterial hypertension, adiposity, hypercholesterolaemia and Type 2 diabetes mellitus. Diabetic nephropathy with nephrosclerosis had also been diagnosed. Other pre-existing disorders were arteriosclerosis in the right subclavian and left carotid artery. The medication she took comprised glimepirid 2 mg, torasemid 10 mg, bisoprolol 10 mg, lisinopril 10 mg, pravastatin 20 mg and acetylsalicylic acid 100 mg.

She mentioned being a habitual smoker (about 40 pack years).

**Figure 2.**
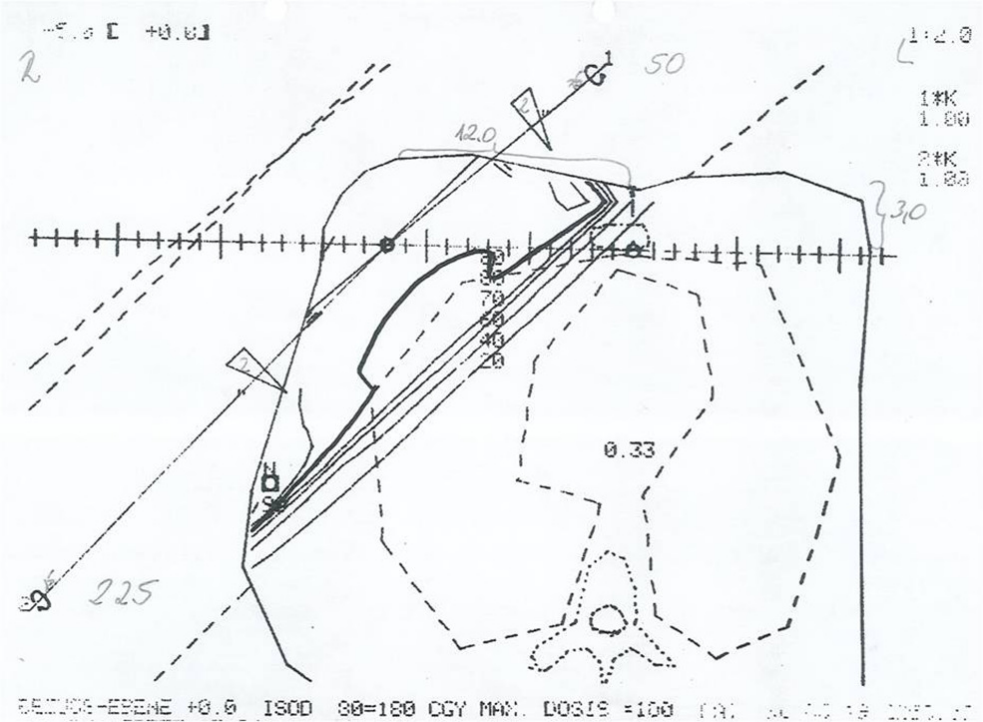
Postoperative irradiation of the right breast with a ^60^Co unit after breast-conserving surgery 20 years earlier. Treatment plan with the dose distribution at the plane containing the isocentre. Relative isodose lines are given (20, 40, 60, 70, 80 and 90%). For simulation, the position of the isocentre in relation to the sternum and the skin is given (handwritten). With a tangential field arrangement, the entire left breast and thoracic wall received 48.6 Gy.

She was diagnosed with a poorly differentiated pT1c pN0 ductal carcinoma of the right breast lateral to the nipple when she was 50 years old. She underwent lumpectomy and axillary dissection. As adjuvant treatment, she received radiation with a ^60^Co unit. With a tangential field arrangement, the entire right breast and chest wall received a total dose of 48.6 Gy, with a daily single dose of 1.8 Gy (Figure 2). Sequentially, the tumour region was additionally treated with 10.8 Gy normofractionated boost irradiation (one field from 0°, size 8 × 6 cm). No relevant acute radiation toxicities were documented.

6 years later, at the age of 56, a locally advanced tumour was diagnosed on the contralateral left side. Mastectomy and dissection of the left axilla revealed a pT4 pN0 ductal carcinoma grade III. Because of infiltration of the pectoralis major muscle, adjuvant radiotherapy was applied with a linear accelerator (6 megavoltage [MV] photons). The chest wall received a normofractionated treatment with 2 Gy single dose up to a total dose of 50 Gy with a tangential field arrangement, the supraclavicular lymph node region with a single field from 330°. Only transient skin toxicities (erythema, hyperpigmentation and dry desquamation) were documented. As the tumour tissue expressed oestrogen and progesterone receptors, the patient underwent tamoxifen as adjuvant antihormonal treatment for 3 years.

At the age of 70 years, the patient was diagnosed with a bronchial carcinoma (pT2 cN0 cM0) in the left upper lobe. Histological findings confirmed a squamous cell carcinoma grade II, which could be removed by wedge resection. In the staging CT scans, multiple heterotopic calcifications in the right breast and thoracic wall were described. As adjuvant therapy, she received five cycles of chemotherapy with carboplatin and gemcitabine.

It was in the course of this chemotherapy that the ulceration in the right mammary fold mentioned above began and gradually started to grow. The formation of the calculus in her right breast had begun years ago. She could not tell us exactly when.

## Differential diagnosis

A differential diagnosis of a condition like this includes a late relapse of the right breast cancer and long-term radiation sequelae.

## Investigations/imaging findings

In order to exclude any progress of one of the patient’s malignant diseases, a restaging took place using bone scan, CT scans of the thorax and ultrasound of the abdomen. No metastases were found.

**Figure 3.**
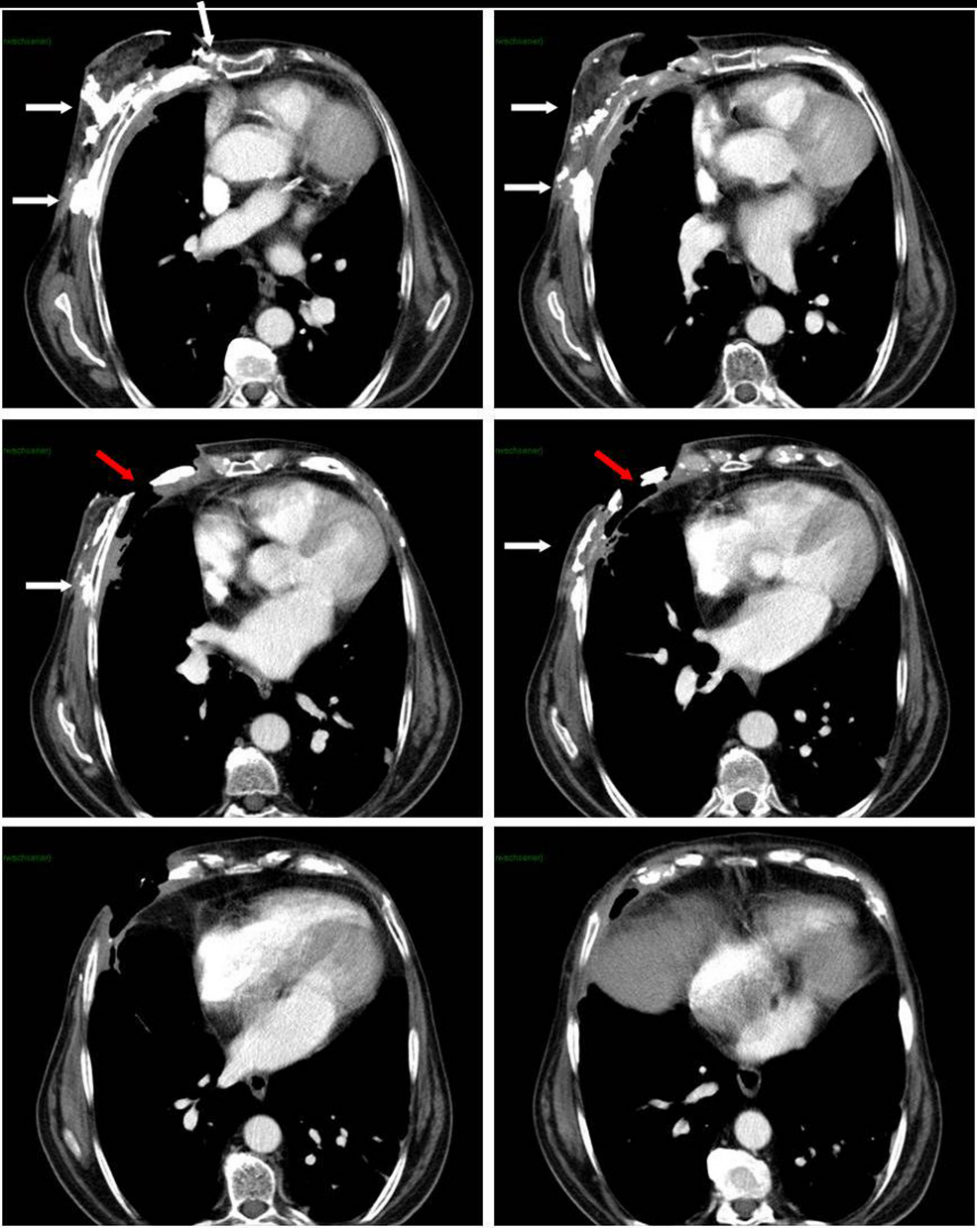
CT scan of the thorax after resection of necrotic and infected tissues: it shows a deep ulceration in the right breast extending to the pleura, remnants of a debruised fourth rib and heterotopic calcifications throughout the breast and thoracic wall tissues. White arrows indicate heterotopic ulcerations, red arrows a debruised rib.

The CT scan of the thorax not only displayed a deep ulceration in the right breast extending to the pleura but also remnants of a debruised fourth rib (Figure 3). The breast and thoracic wall tissues were permeated with profuse heterotopic calcifications. Pulmonary or lymphatic metastases were not detected.

A biopsy was taken and revealed lipoatrophic mammary glands, chronically recurring inflammation and a gangrenous process. In addition, prominent secondary calcifications and ossifications became visible. However, there was no sign of any malignant cells.

These findings confirmed the diagnosis of a non-malignant ulceration, probably as a consequence of the radiation therapy applied 21 years earlier.

## Treatment

The patient was treated with systemic antibiotics (cefuroxim). The wound was regularly debrided. As soon as her temperature decreased and her general condition improved, she was transferred to the thoracic surgery department.

Under a general anaesthetic, the margins of the wound were carefully excised to cut out the infected tissues. For a month, the wound was cleaned and bandaged, and she received vacuum-assisted sealing therapy in order to stop the inflammation.

**Figure 4.**
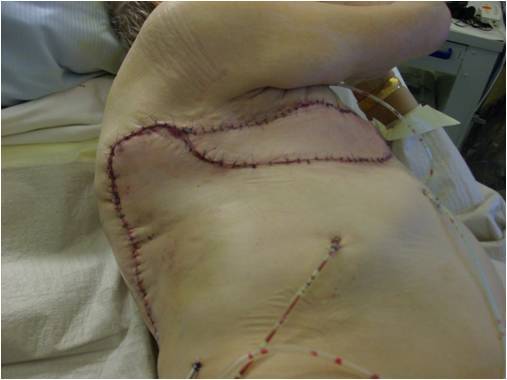
Postoperative aspect after resection of the remaining breast tissue and the destructed ribs. For plastic reconstruction, a latissimus dorsi flap with covering skin was successfully implanted.

After that, the patient underwent plastic surgery in the course of which the remaining breast tissues and the decomposed fourth, fifth and sternal part of the sixth rib were resected. This necessitated a ligation of the internal thoracic artery. A latissimus dorsi flap with the covering skin was created and successfully rotated into the right thoracic wall (Figure 4).

## Outcome and follow-up

As there was virtually no further soft tissue at the medial side of the flap, a wound healing disorder occurred postoperatively in this region. It took months of regular, daily wound cleaning and dry bandages until granulation tissue led to secondary wound healing.

**Figure 5.**
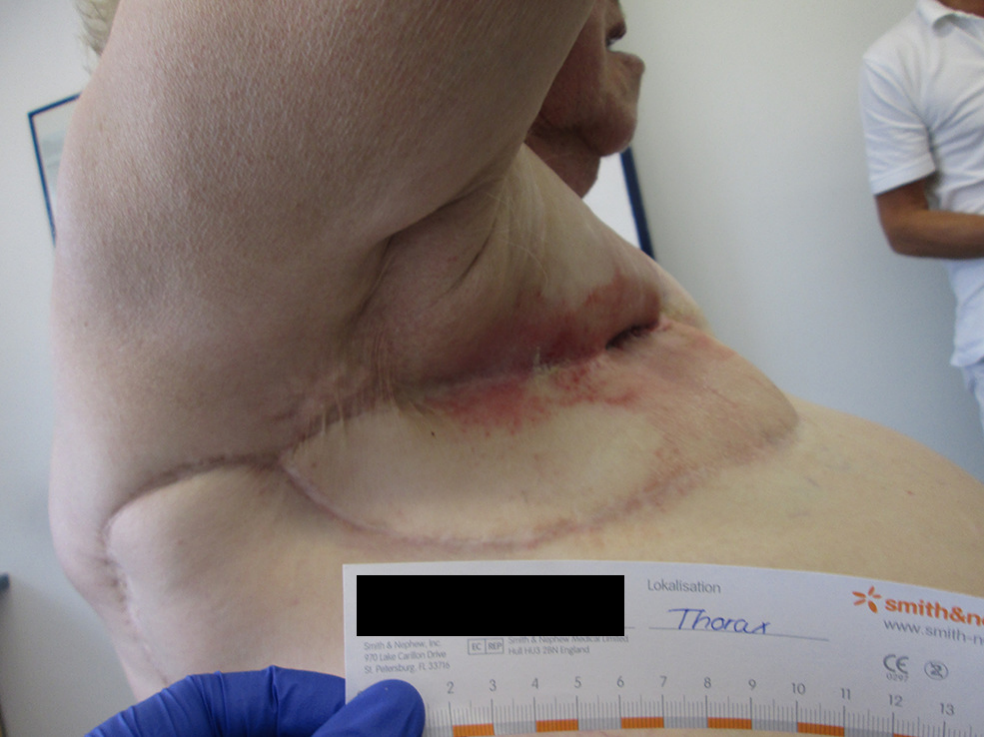
Clinical aspect about 2 years after plastic surgery. The patient had fully recovered. Owing to dermatomycosis, she received a topical antimycotic.

2 years later, the wound had healed. A fungal infection of the skin was treated with clotrimazol. She did not feel pain in her scars. She was still without any evidence of a relapse or metastases of her malignant diseases (Figure 5).

## Learning points 

Late radiation sequelae of the breast, such as fibrosis, calcification, ulceration and osteoradionecrosis, are rarely observed in modern radiotherapy planning and treatment. Patients at highest risk are those treated with radiation field overlaps (thus creating hot spots) or with long-term survival after high radiation doses—especially in the orthovolt and cobalt eras.^1,2^ It can be assumed that low MV energies increase the risk of rib fractures and tissue necrosis. Rib fractures commonly occur in the anterior aspects of the third to fifth rib.^3^ In a large patient series (*n  *= 1624) from Havard Medical School, rib fractures occurred in 1.8% of patientsat a median time of 12 months after treatment.^4^ 2.2% of the patients treated with 4 MV irradiation experienced fractures in comparison to 0.4% after 6 or 8 MV (*p *= 0.05). In this study, only 0.18% of the patients developed tissue necrosis, requiring surgical intervention between 22 and 114 months after irradiation.

As cobalt irradiation emits photons with a maximal energy of about 1.3 MV, these sequelae may occur even more frequently. The affected side of our patient was treated with a ^60^Co unit. The region of ulceration was not related to any field arrangements, as the boost volume was located more cranially than the ulceration. However, only rudimental treatment planning was available at that time. The dose distribution was estimated only in the plane containing the isocentre. This caused a risk of unintended hot spots in other areas of the treatment volume. A study on dosimetric aspects of ^60^Co irradiation showed dose maxima up to 125% of the prescribed dose in the anterior region of the inferior aspect of the breast—exactly in the same region in which our patient developed ulceration about 20 years later.^5^

Interestingly enough, our patient did not develop any post-radiation sequelae on her left side, which had been treated with 6 MV photons and a more sophisticated CT planning 6 years later. This finding makes an increased radiosensitivity of the patient very unlikely.

Concomitant diseases may play a crucial role in the development of radiation sequelae. Common consequences of diabetes are microvascular occlusive changes, leading to impaired vascularity. Capillary hyalinization, arteriolar obliteration and arteriosclerosis with resultant tissue hypoxia are regularly found.^6^ Furthermore, arterial hypertension as well as smoking habits may lead to an increase in acute and late side effects of curative radiotherapy in various anatomical regions.^6^

Chemotherapy apparently seems to increase the risk of developing miscellaneous late toxicities after radiotherapy of breast cancer.^7,8^ It is noteworthy that our patient developed ulceration 20 years after irradiation while undergoing chemotherapy because of lung cancer. It seems reasonable to assume that decomposition and disintegration of soft and bone tissues were eventually triggered off by chemotherapy. This could have been caused by directly damaging the tissues or indirectly via immunosuppression.

Subcutaneous heterotopic calcifications such as radiation sequelae are relatively rare and have been infrequently described in various publications.^9^ The largest number of patients (*n *= 15) has been reported by Carl and Hartmann.^10^ These patients received various radiation techniques, types and fractionation schedules in the median 19 years (range 2–31 years) before diagnosis of chronic sequelae. As in our case, calcifications were linked with other severe side effects in the surrounding tissues, such as fibrotic changes and ulcerations.

In short, in our patient, numerous risk factors such as the development of post-radiogenic fibrosis, ulceration and heterotopic calcification interacted. Vascular damages caused by diabetes, arterial hypertension and smoking could have aggravated a possible—though not proven—unintended overdosage of ^60^Co irradiation. All this resulted in severe tissue damage. In the end, the breakdown of tissue integrity was triggered by chemotherapy of bronchial carcinoma.

The only therapeutic option to treat the superinfected vast ulceration was a surgical removal of the fibrotic and hypoxic tissues, and the closure of the wound by implantation of a latissimus dorsi flap with independent vascularization.
